# Two Silicea Cases in One

**Published:** 1883-09

**Authors:** Charles F. Millspaugh

**Affiliations:** Binghamton, N. Y.


### CLINICAL BUREAU.

### TWO SILICEA CASES IN ONE.

CHAS. F. MILLSPAUGH, M. D., BINGHAMTON, N. Y.

It is so seldom that two patients are met with at the same time affected exactly alike, each giving, in the main, symptom for symptom, that I am led to publish from my case-book the following notes. Allow me here to suggest that the reader should underscore in his Materia Medica the symptoms given, as some are not especially reproven and italicized in the *Encyclopædia*, while several prominent symptoms which I have marked with an * are not found, so far as I have observed, in any proving.

1. Mrs. G.—Age 35, very light complexion. Had suffered from cephalalgia since the age of 9. To give her own words, "I have not passed a week without a headache in twenty-five years."

2. Mrs. M.—Age 28, complexion the same. Had suffered the same as Mrs G. since the age of 10.

The first measure I adopted in each case, I find, was to prohibit the use of tea, and to give China$^{30}$, two doses a week apart, to antidote the habit and its effects: after this I gave each case a dose of Silicea$^{1m}$, B. & T. This is all the medicine used, and no headaches have occurred since (fifteen months) except following excessive eating of pastry or great overexertion, when other symptoms and remedies to suit were given. These exceptions have been very few.

Symptoms presented and cured:

(7) Sensitiveness to noise.[1 2]

(8) Very sensitive, even to weeping.[2]

(43) Everything frets her and makes her peevish.[1 2]

(79) Vertigo, on rising up.[1 2]

(113) Her head swam as if she were drunk, compelled her to sit down.[1 2]

(*) Yawning preceded headaches.[1 2]

(184) Tearing pain as if the head would burst, with deep and superficial throbbing, chilliness; she was obliged to tie up her head tightly and lie down.[1 2]

(211) A bruised pain above the eyes, first affecting the left side, then passing over to the right, and remaining in both, spreading thence over the whole head < by opening the eyes.[1 2]

(253) Violent pressive pain in the left temple.[1 2]

(263) Bruised pain on the vertex.[1 2]

(294, 295) Excessive tenderness of the scalp, which became so sore she could not brush her hair.[1 2]

(*) Nausea with chilliness during the headache.[1]

(325) Small severe pressive pain over the left eye.[1 2]

(359) Paroxysmal blindness of the eyes in daylight.[1]

(368) Black before the eyes after the headache.[1]

(This symptom with No. 184 led me to think of Silicea before the other symptoms were given.)

(550) Great dryness of the mouth without thirst.[1 2]

(688) Very frequent nausea without vomiting and not affecting the appetite or taste.

(*) Dilated pupils during headache.[1 2]

(1009) Menses increased with great coldness of the whole body.[1 2]

(1096) Oppression of the chest without pain, with inability to take a long breath.[1 2]

(1214) Pain in small of back as if beaten (1215) especially at night.[1 2]

Great weakness and weariness.[1 2]

(1519) Cramps in the soles of the feet.[1 2]

(1570) Emaciated.[1 2]

(1605) Bruised sensation over the whole body at night, as if had lain in a cramped position.[1 2]

(1616) Sensation as of knives cutting into her flesh point first.[2]

(1700) Constant inclination to yawn.[1 2]

(1710) Great sleepiness early in the evening.[1 2]

(1793) She dreams many times when sleeping off a headache of being seized by the fingers.[1]

(*) Dreams of clasping her fingers so tightly as to cause great suffering, which awoke her.[1]

(1797) Every night after a headache for some months she dreams of being choked by a grasping hand. No body accompanied it.[2]

(1814) Constant chilliness preceding headache with occasional flashes of heat.[1 2]

(1850) Icy cold feet constantly in the evening as soon as the stockings were removed,[1 2] (1854) preventing sleep,[1 2] (1855) until they became warm, as they usually did about a half hour after retiring.[2]

(906) Hard, lumpy stools,[1 2] (921) which were usually of 3 days accumulation,[1 2] (919) voided only after many frequent calls and much straining, leaving a very unsatisfactory feeling in the rectum.[2]

It seems to me that in these cases and in other Silicea headaches continuing from early life which have came under my notice, the pathology must be something in connection with the tissues themselves. I am inclined to think the trouble organic, rather than functional; and a great part of its action must be expended upon the nerve centres generally.

Leucophlegmatic females who have suffered from headaches since some severe disease of youth, like scarlet fever, diphtheria, croup, or adenitis, will give symptoms of Silicea oftener than of any other drug.

